# Combined transhepatic and transsplenic recanalization of chronic splenic vein occlusion to treat left-sided portal hypertension: A cases report

**DOI:** 10.1097/MD.0000000000037109

**Published:** 2023-02-02

**Authors:** Jie Liang, Xu Feng, Min Peng, Jin-Tao Duan, Yao-Yong Chen, Jun Zhu

**Affiliations:** a School of Clinical Medicine, Chengdu Medical College, Chengdu, Sichuan Province, China; b Department of Interventional, The Second People’s Hospital of Yibin, Yibin, Sichuan Province, China.

**Keywords:** angioplasty, left-sided portal hypertension, splenic vein, stenting

## Abstract

**Rationale:**

This report describes a unique case of a combination transhepatic and transsplenic recanalization of chronic splenic vein occlusion to treat left-sided portal hypertension (LSPH).

**Patient concerns:**

In this case report, we report a 49-year-old male who was admitted due to LSPH causing black stools for 2 days and vomiting blood for 1 hour.

**Diagnoses:**

The patient has a history of multiple episodes of pancreatitis in the past. After admission, abdominal contrast-enhanced CT scan showed the appearance of pancreatitis, with extensive splenic vein occlusion and accompanied by gastric varicose veins, indicating the formation of LSPH.

**Intervention:**

The patient received treatment with a combination of splenic and hepatic splenic venoplasty.

**Outcomes:**

Follow up for 1 year, CT and gastroscopy showed disappearance of gastric varices.

**Lessons:**

Splenic venoplasty is an effective method for treating LSPH. When it is difficult to pass through the occluded segment of the splenic vein through a single approach, percutaneous double approach splenic venoplasty can be attempted for treatment.

## 1. Introduction

Left-sided portal hypertension (LSPH) is a special type of extrahepatic portal hypertension, which is relatively rare in clinical practice and accounts for about 5% of all portal hypertension.^[1]^ Among them, bleeding from gastric varices is the most serious complication, posing a serious threat to the patient life. At present, the main treatment method for LSPH is splenectomy. Surgical excision, however, is an invasive procedure that requires general anesthesia and a relatively long hospital stay. Splenic venoplasty, as a minimally invasive interventional treatment method, can restore normal vascular anatomy while preserving spleen function, making it an alternative method for splenectomy.^[2]^ However, the success rate of splenic venoplasty is relatively low, especially for some cases of long segment splenic vein occlusion caused by chronic pancreatitis. It is difficult to use only a single portal vein, jugular vein, or splenic vein approach for recanalization. In this study, we report a cases of LSPH with gastric varices in which percutaneous intervention with a combined transhepatic and transsplenic approach was successful.

## 2. Case report

A 49-year-old male was admitted to the hospital for 2 days of relieving black stools and 1 hour of vomiting blood, with a blood volume of approximately 200 mL. The patient had a history of pancreatitis for many times and diabetes for half a year.

**Figure 1.**
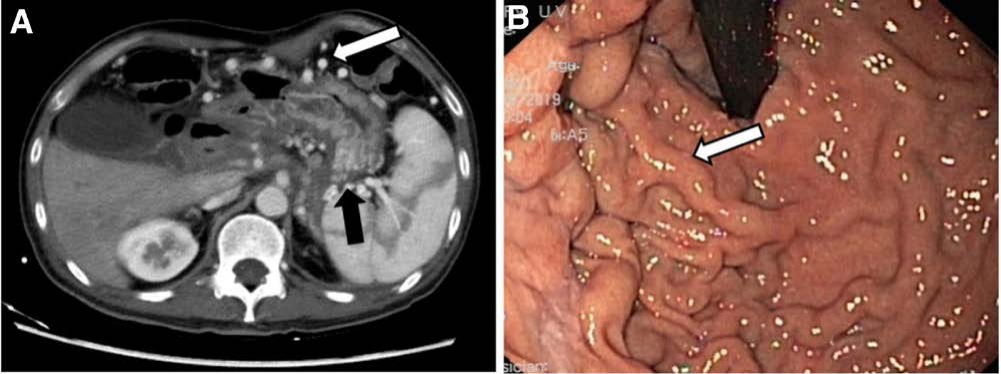
Abdominal enhanced CT and endoscopic images prior to splenic venoplasty. (A) Axial contrast enhanced CT abdomen at the level of renal hilum venous stage showed tortuosity and dilatation of fundus gastric veins (black arrow) and gastroepiploic veins (white arrow), and unclear splenic veins. (B) Endoscopic images showing severe varicose gastric fundus veins (white arrow).

On admission, abdominal contrast-enhanced CT showed blurred pancreatic structure, swelling of surrounding fascia, extensive occlusion of splenic veins, and tortuous expansion of gastric veins (Fig. 1A), indicating PSPH. Endoscopic examination revealed severe esophageal varices, accompanied by varices in the fundus and body of the stomach (Fig. 1B). Endoscopic sclerotherapy was performed, but the hemostatic effect was ineffective.

After multidisciplinary consultation and discussion, it was considered that the long-term chronic pancreatitis of this patient resulted in heavy adhesion around the pancreas and spleen, and the splenectomy had a greater risk of bleeding, so it was not considered. Therefore, we plan to perform splenic venoplasty. Due to the long splenic vein occlusion, it is expected that it will be difficult to perform splenic venoplasty using only a single transhepatic vein route. Hence, we prepared a combined transhepatic and transsplenic approach. Informed consent was obtained from the patient.

**Figure 2.**
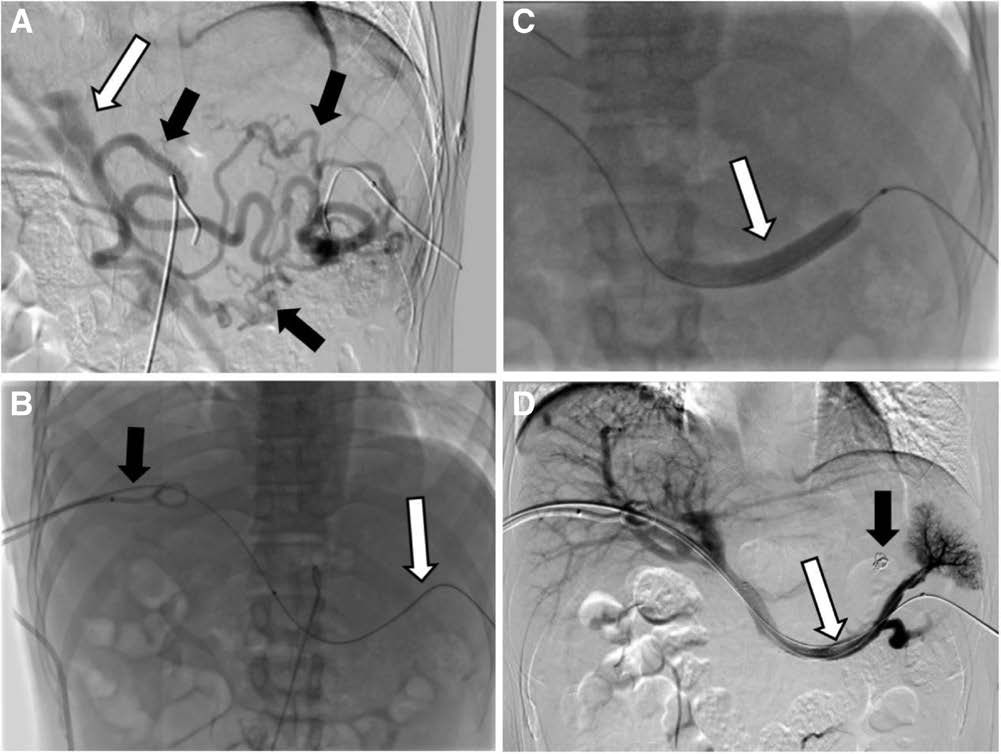
A 49-year-old male underwent percutaneous dual approach splenic venoplasty due to LSPH. (A) Under the guidance of DSA and ultrasound, percutaneous puncture of the inferior splenic vein was performed and a guide wire catheter was inserted. The splenic venography showed no visualization of the main splenic vein, while the gastric omental vein and surrounding collateral vessels (black arrow) showed no visualization and flowed back to the portal vein (white arrow); (B) the guide wire catheter was used to explore the splenic vein through the splenic puncture pathway and successfully passed through the narrow segment of the splenic vein (white arrow). An 8F long sheath was introduced into the portal vein puncture pathway to grasp the tip of the guide wire and pull it out (black arrow); (C) introduce 4, 6, and 8 mm balloon catheters from the portal vein puncture pathway through a penetrating guide wire to dilate the splenic vein occlusion segment (white arrow); (D) implant 10 mm × 8 cm, 12 mm × 8 cm vascular stent and 10mm balloon catheter were introduced for stent dilation. The splenic vein system was reimaged to show that the splenic vein had been opened (white arrow), and the steel ring used in the embolization of gastric coronary vein and gastric varicose vein (black arrow) was visible. DSA = digital subtraction angiography, LSPH = left-sided portal hypertension.

Under the guidance of ultrasound and digital subtraction angiography, percutaneous puncture was performed on the right branch of the portal vein and the branch of the splenic vein. A catheter was inserted into the splenic vein branch, and splenic venography showed splenic vein occlusion with the formation of peripheral collateral vessels (Fig. 2A). A guide wire was introduced through the splenic vein occlusion segment (Fig. 2B), and then an 8F long sheath was introduced into the portal vein pathway to grasp the tip of the guide wire and pull it out (to open the channel). Select the coronary vein and posterior vein of the stomach for ultrasound, and inject 2 steel coils to embolize the varicose vein. Then, a balloon catheter is introduced through the portal vein puncture pathway to dilate the splenic vein occlusion segment (Fig. 2C), and a vascular stent is placed and unfolded. The splenic vein system underwent another angiography, which showed that the splenic vein had reopened and the venous reflux of the spleen was restored (Fig. 2D). Finally, the hepatic and splenic pathways were blocked with coils. Anticoagulant therapy was initiated from the second day after stent placement to prevent thrombosis, and there were no postoperative complications related to surgery.

**Figure 3.**
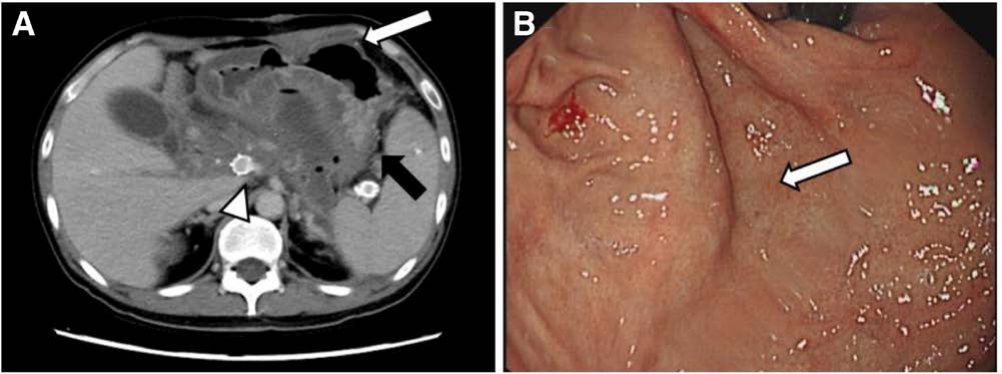
After 1 year of surgery, abdominal enhanced CT and endoscopic imaging were performed. (A) Abdominal contrast-enhanced CT showed disappearance of gastric fundus veins (black arrow) and absence of varicose gastric omentum veins (white arrow), with splenic vein stents unobstructed (arrow); (B) endoscopy shows disappearance of gastric varices (arrow).

During the 1-year follow-up after surgery, there was no recurrence of bleeding, and follow-up CT and endoscopic examination showed the disappearance of gastric varices (Fig. 3A and B).

## 3. Discussion

Splenoplasty can effectively treat gastrointestinal bleeding caused by LSPH. This surgery aims to recanalize the splenic vein through stent placement, thereby restoring splenic vein blood flow and reducing collateral venous pressure. There are currently literature reports on the successful treatment of LSPH bleeding with splenic vein plasty.^[3–5]^ Compared with traditional splenectomy, splenic venoplasty has no surgical complications such as bleeding and infection, and has a shorter hospital stay and relatively lower overall treatment costs.^[6]^ And compared to partial splenic embolization, splenic venoplasty has significant advantages in preventing PSPH rebleeding. The bleeding rate of splenic venoplasty is only 7.1%, which is much lower than partial splenic embolization (rebleeding rate is 47.8%).^[2]^

At present, the main approaches for splenic venoplasty include the transhepatic and transjugular access, both of which are operated through the portal vein to reach the lesion site for treatment. There is some debate in the literature regarding the use of transjugular and transhepatic approaches. Some scholars believe that transhepatic approaches increase the risk of bleeding, but the jugular approach requires the use of transjugular intrahepatic portosystemic shunt technology, which is more complex, and the transhepatic approach is relatively simple.^[2,7]^ The transsplenic approach is another pathway method for treating portal vein system diseases, which can be used for balloon angioplasty, stent placement, or varicose vein embolization through the splenic approach.^[8–10]^ Haddad et al showed that there was no significant difference in bleeding complications between transsplenic and transhepatic access for portal venous interventions (3/24 [12.5%] transsplenic vs 10/124 [8.1%] transhepatic; *P* = .44).^[11]^Therefore, splenic vein recanalization through the transsplenic approach is feasible. However, the most serious complication of the transsplenic approach is bleeding at the puncture site. To control bleeding, it is necessary to block the puncture site.^[12]^ In this case, we used spring coils to block the splenic and hepatic vein puncture sites after surgery to prevent bleeding, and there was no bleeding at the puncture site during postoperative follow-up.

The application of splenic vein stenting might be limited by the low success rate, previous studies have reported that the success rate of splenic vein stent implantation is only 53.8%.^[2]^ The main reason for the failure of splenic vein plasty is the inability to pass through the obstructed segment of the splenic vein,^[2]^ especially the long segment splenic vein obstruction caused by chronic pancreatitis, which makes it difficult to pass through the narrow segment of the splenic vein. In cases where it is difficult to open the narrow segment of the splenic vein through the portal vein approach, increasing the splenic vein puncture approach may increase the probability of reopening the obstructed segment of the splenic vein. This patient example successfully demonstrates the feasibility of percutaneous dual approach splenic vein plasty for the treatment of LSPH with bleeding. At present, there is no unified standard for the treatment of LSPH. Therefore, percutaneous dual approach splenic venoplasty can be used as another method to improve the success rate of splenic venoplasty as it is difficult to open the splenic vein through a single approach.

## Author contributions

**Conceptualization:** Xu Feng.

**Data curation:** Min Peng, Jin-Tao Duan.

**Methodology:** Yao-Yong Chen.

**Supervision:** Jun Zhu.

**Writing – original draft:** Jie Liang.

**Writing – review & editing:** Jun Zhu.
